# Supplementary material for: Development of a reliable, sensitive, and convenient assay for the discovery of new eIF5A hypusination inhibitors
Source: PLoS One. 2025 Feb 12;20(2):e0308049. doi: 10.1371/journal.pone.0308049 (PMC11819603; doi:10.1371/journal.pone.0308049)
Supplement: S1 Fig — (DOCX) [file pone.0308049.s001.docx]

Figure S1. Bacterial construction of recombinant human eIF5A, DHPS or DOHH proteins and corresponding elution profile of column-purified proteins analysed by SDS-PAGE and stained with Coomassie blue.
