# Supplementary figures and images for: Development of a reliable, sensitive, and convenient assay for the discovery of new eIF5A hypusination inhibitors
Source: PLoS One. 2025 Feb 12;20(2):e0308049. doi: 10.1371/journal.pone.0308049 (PMC11819603; doi:10.1371/journal.pone.0308049)

Figure S2.Reaction of hypusination of eIF5A performed at various pH.


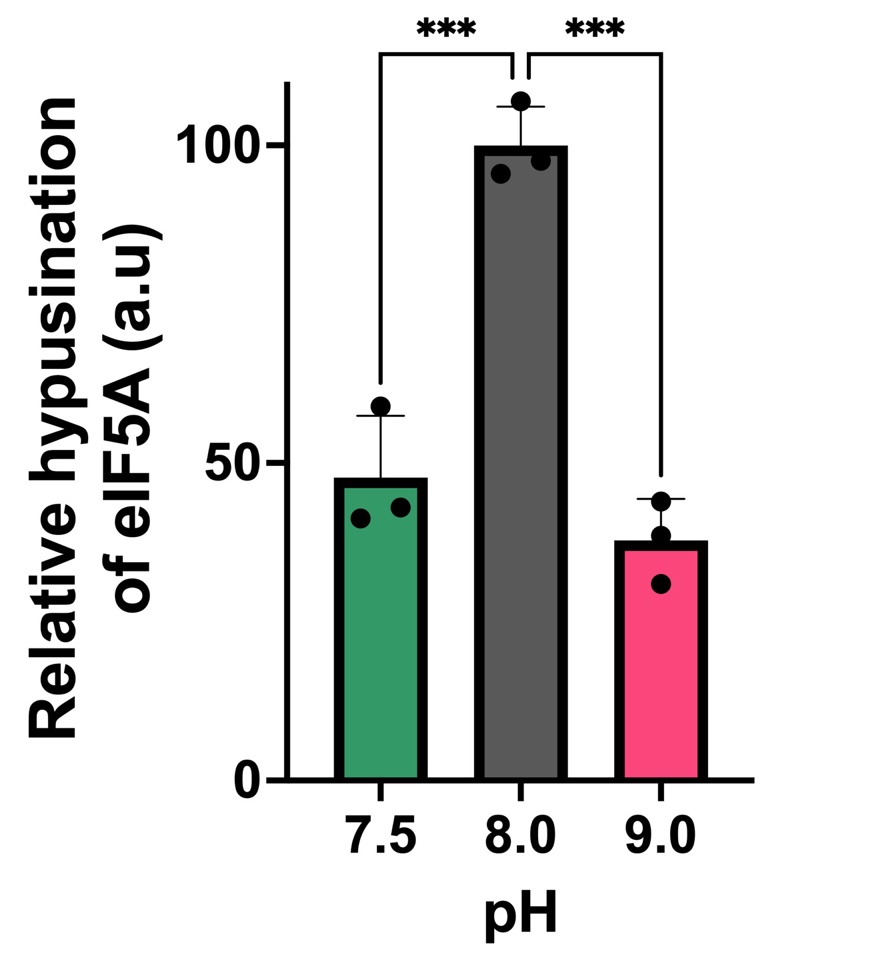

Supplement: S2 Fig — (DOCX) [file pone.0308049.s002.docx]

Figure S4. 1H NMR spectrum (400 MHz, methanol-*d4*) of *N*,*N*'-di-*tert*-butoxycarbonyl-4-hydroxybutylguanidine (**5**).


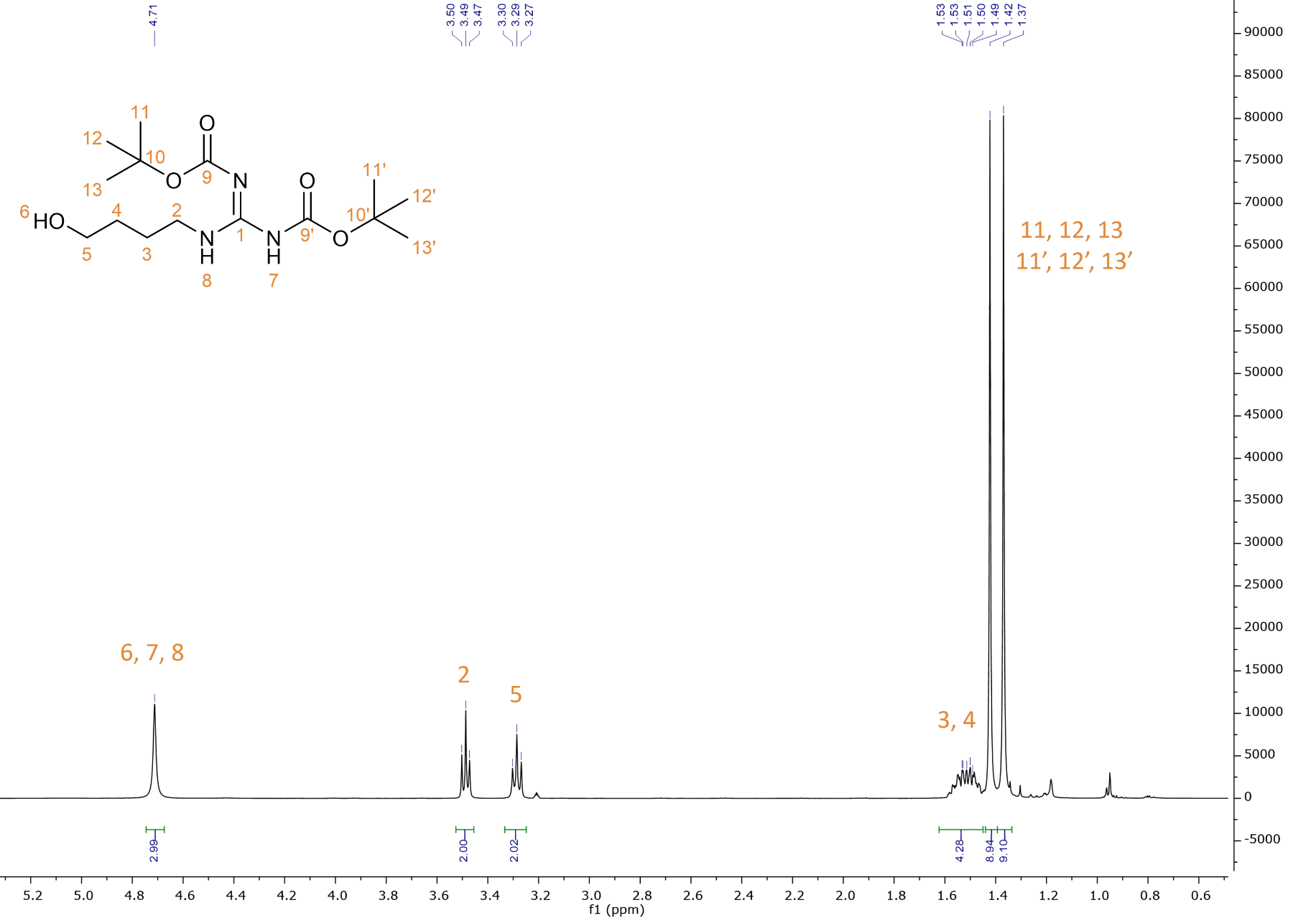

Supplement: S4 Fig — (DOCX) [file pone.0308049.s004.docx]

Figure S5. 13C NMR spectrum (101 MHz, methanol-d4) of *N*,*N*'-di-*tert*-butoxycarbonyl-4-hydroxybutylguanidine (**5**).


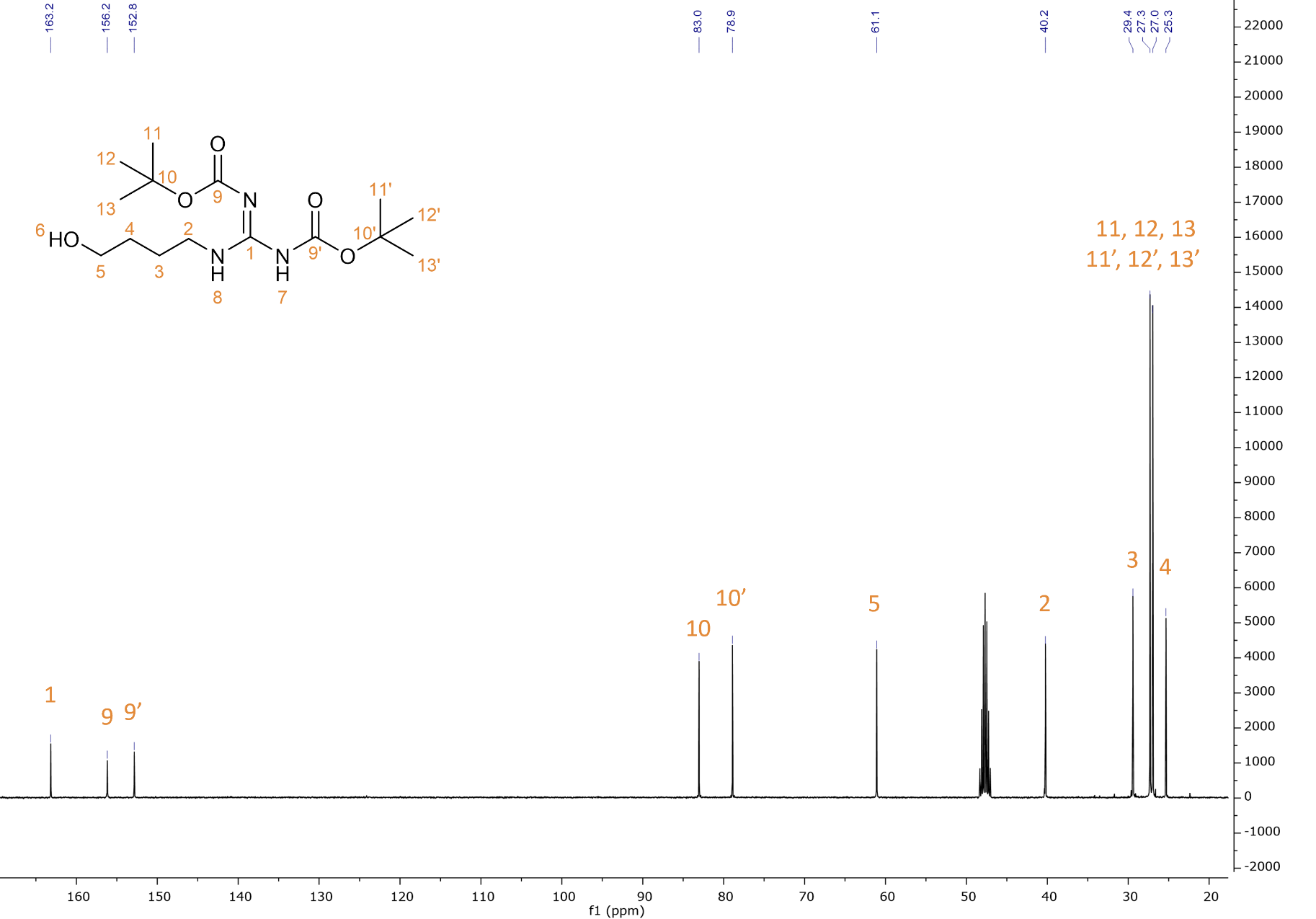

Supplement: S5 Fig — (DOCX) [file pone.0308049.s005.docx]

Figure S6. 1H NMR spectrum (400 MHz, methanol-*d4*) of 4-hydroxybutylguanidine (**1**).


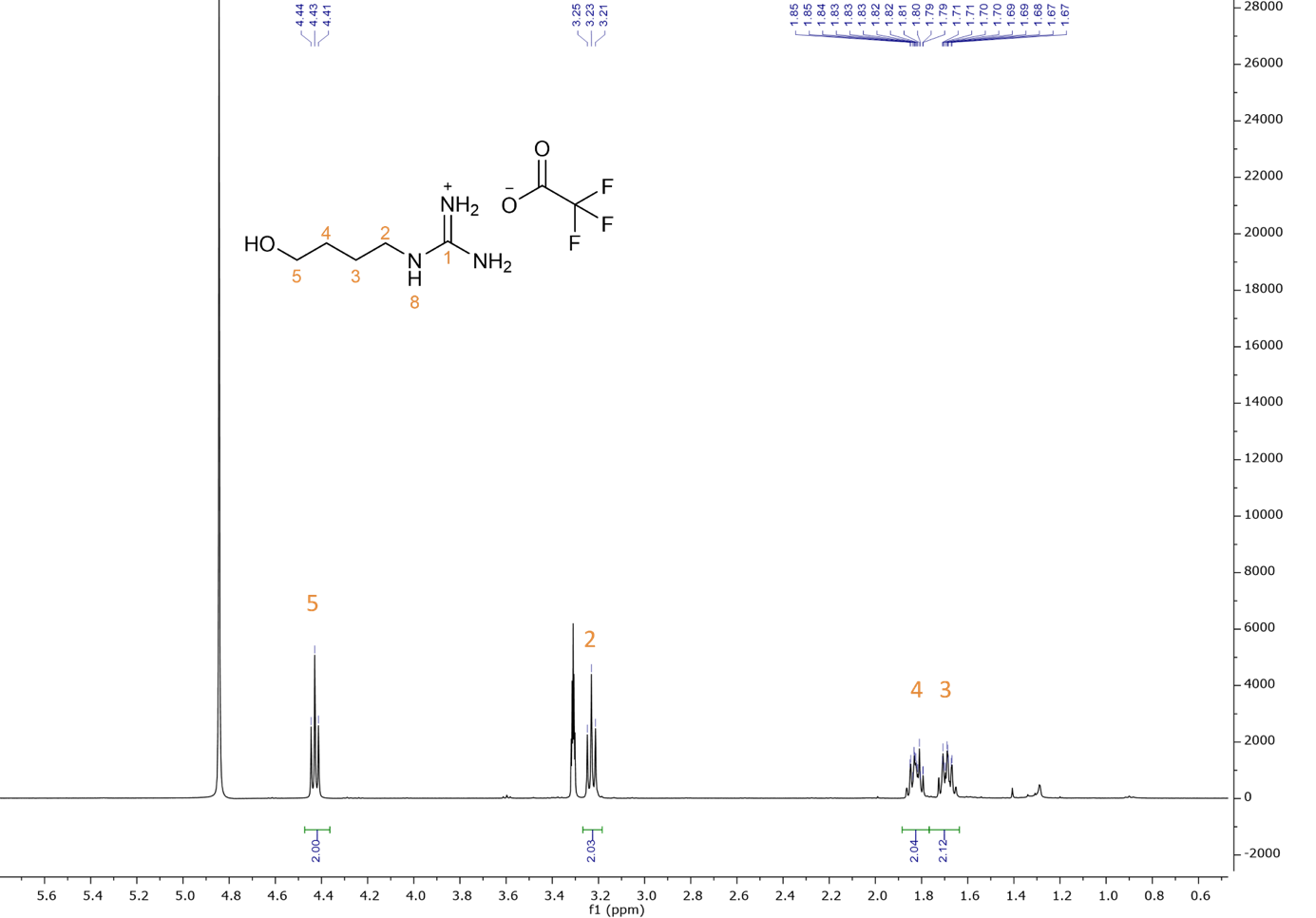

Supplement: S6 Fig — (DOCX) [file pone.0308049.s006.docx]

Figure S7. 13C NMR spectrum (101 MHz, methanol-*d4*) of 4-hydroxybutylguanidine (**1**).


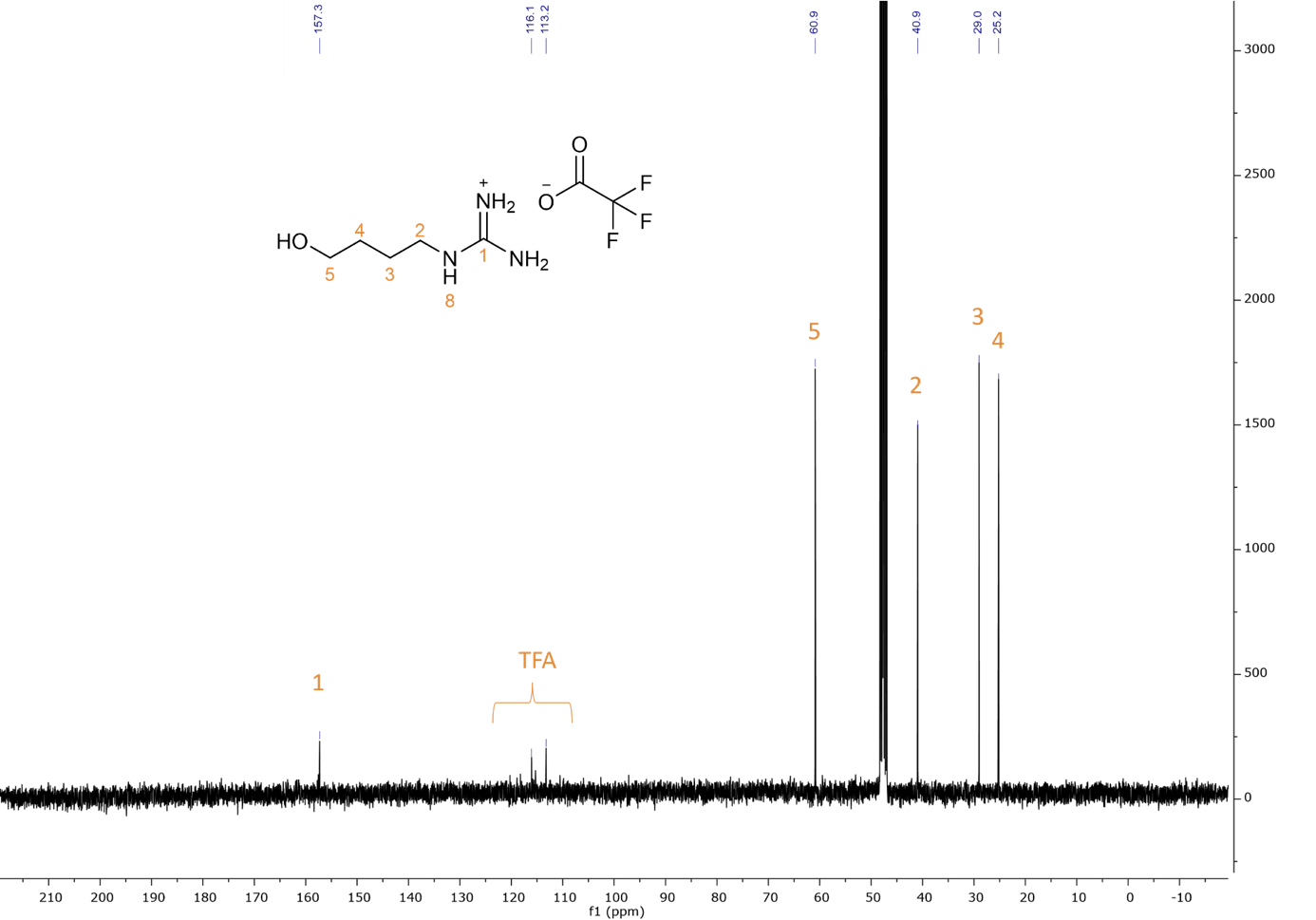

Supplement: S7 Fig — (DOCX) [file pone.0308049.s007.docx]

Figure S8. 1H NMR spectrum (400 MHz, methanol-*d4*) of *N*,*N'*-di-*tert*-butoxycarbonyl-6-hydroxyhexylguanidine (**6**).


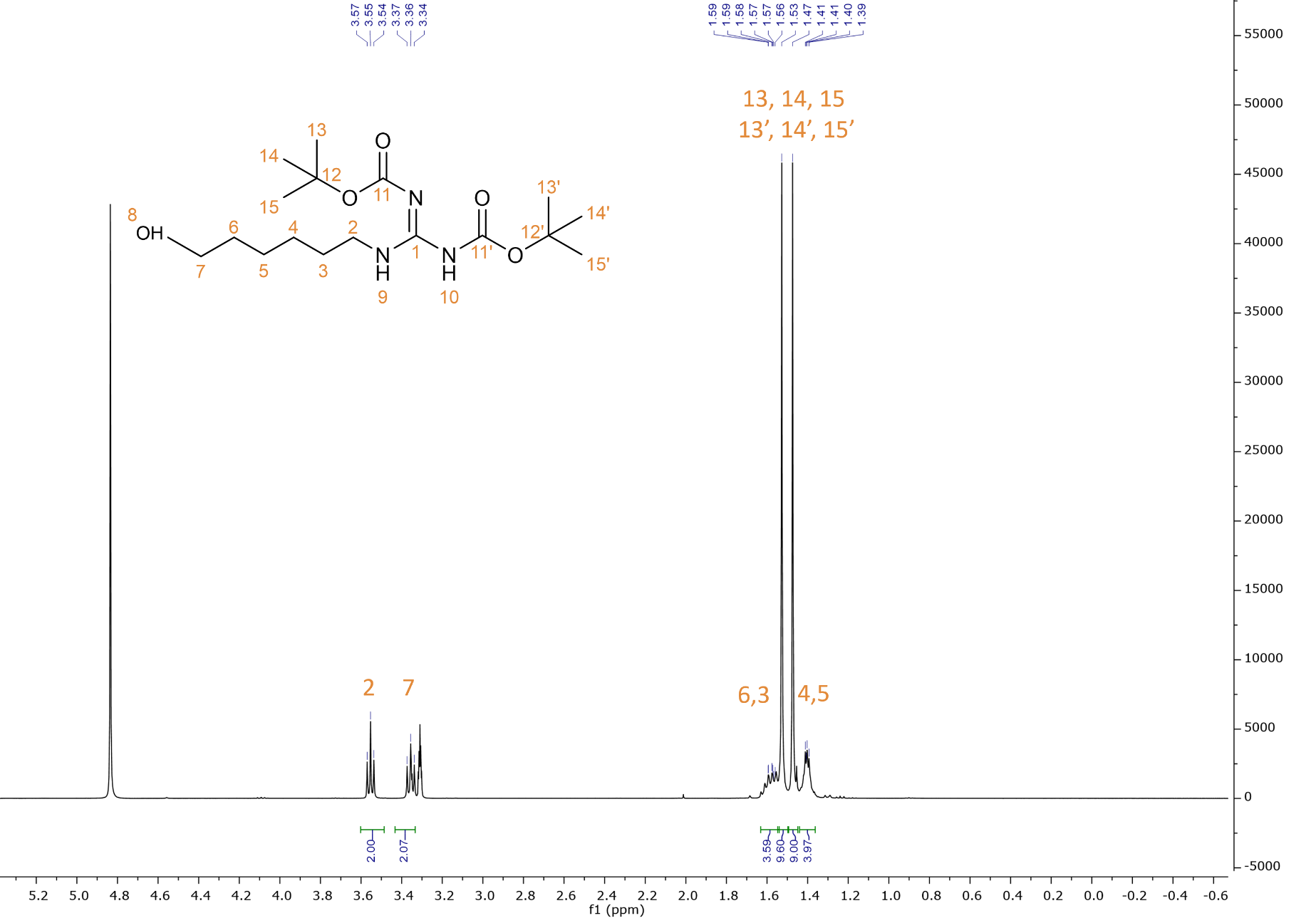

Supplement: S8 Fig — (DOCX) [file pone.0308049.s008.docx]

Figure S9. 13C NMR spectrum (101 MHz, methanol-*d4*) of *N*,*N*’-di-*tert*-butoxycarbonyl-6-hydroxyhexylguanidine (**6**).


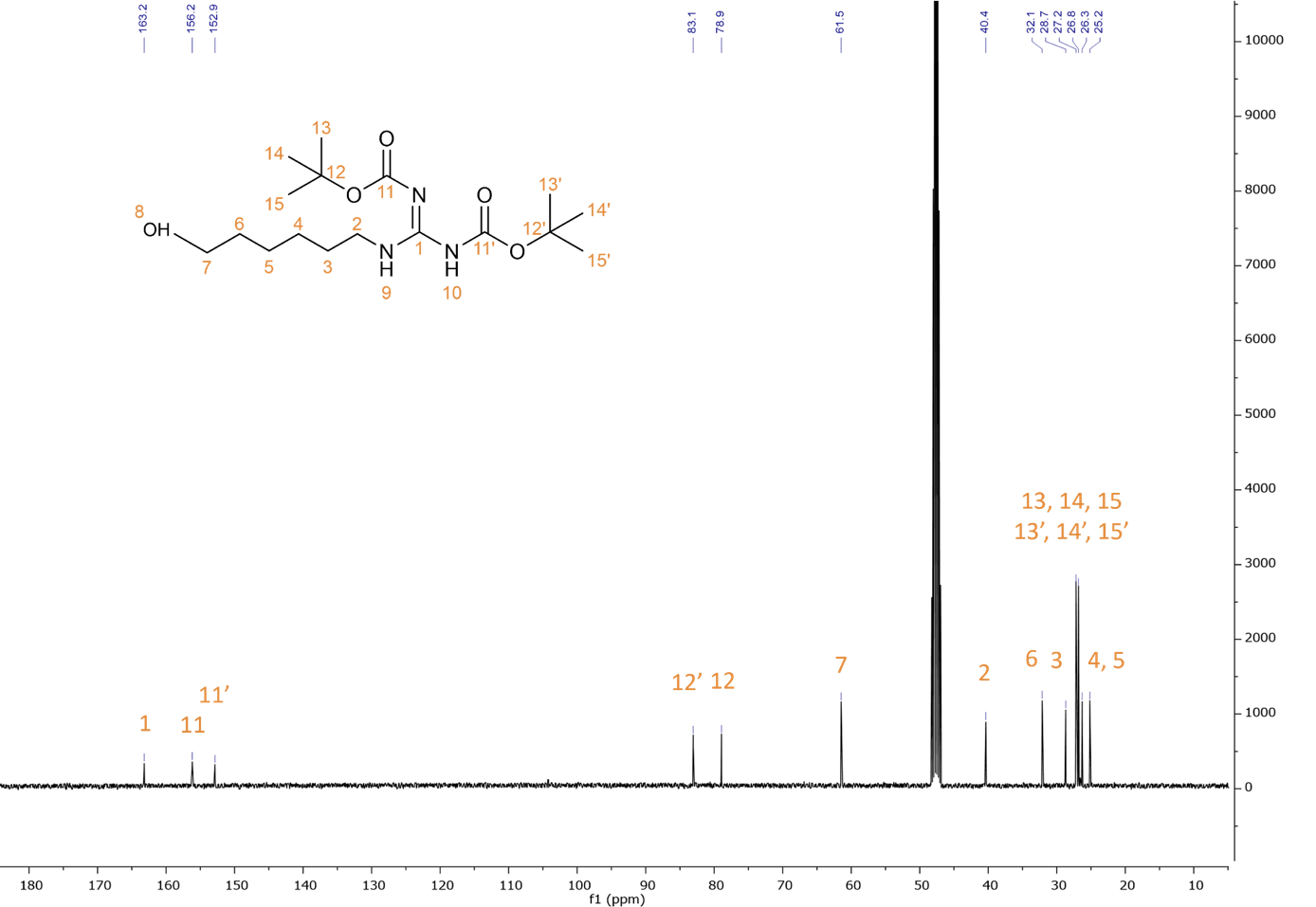

Supplement: S9 Fig — (DOCX) [file pone.0308049.s009.docx]

Figure S10. 1H NMR spectrum (400 MHz, methanol-*d4*) of 6-hydroxyhexylguanidine (**2**).


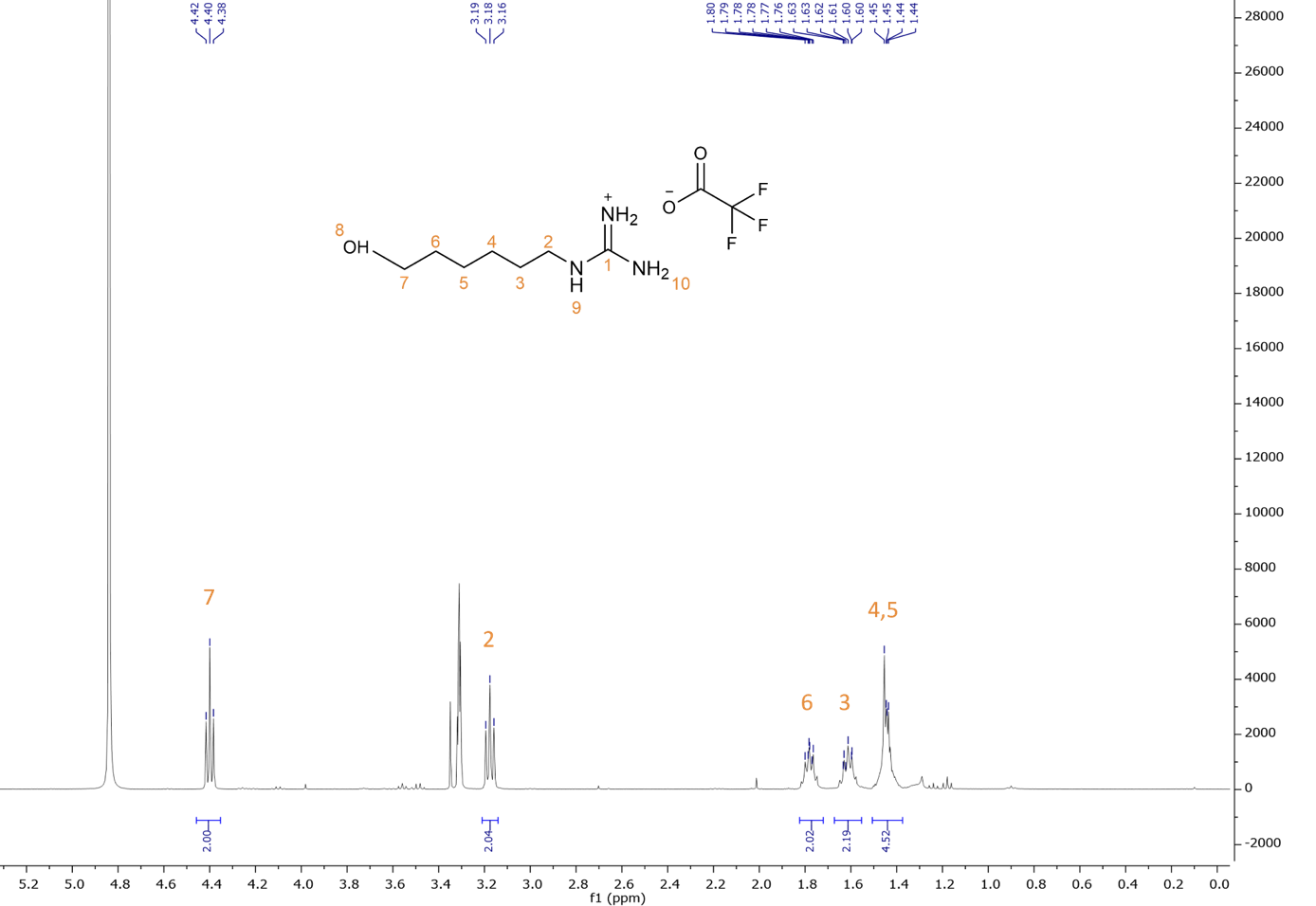

Supplement: S10 Fig — (DOCX) [file pone.0308049.s010.docx]

Figure S11. 13C NMR spectrum (101 MHz, methanol-*d4*) of 6-hydroxyhexylguanidine (**2**).


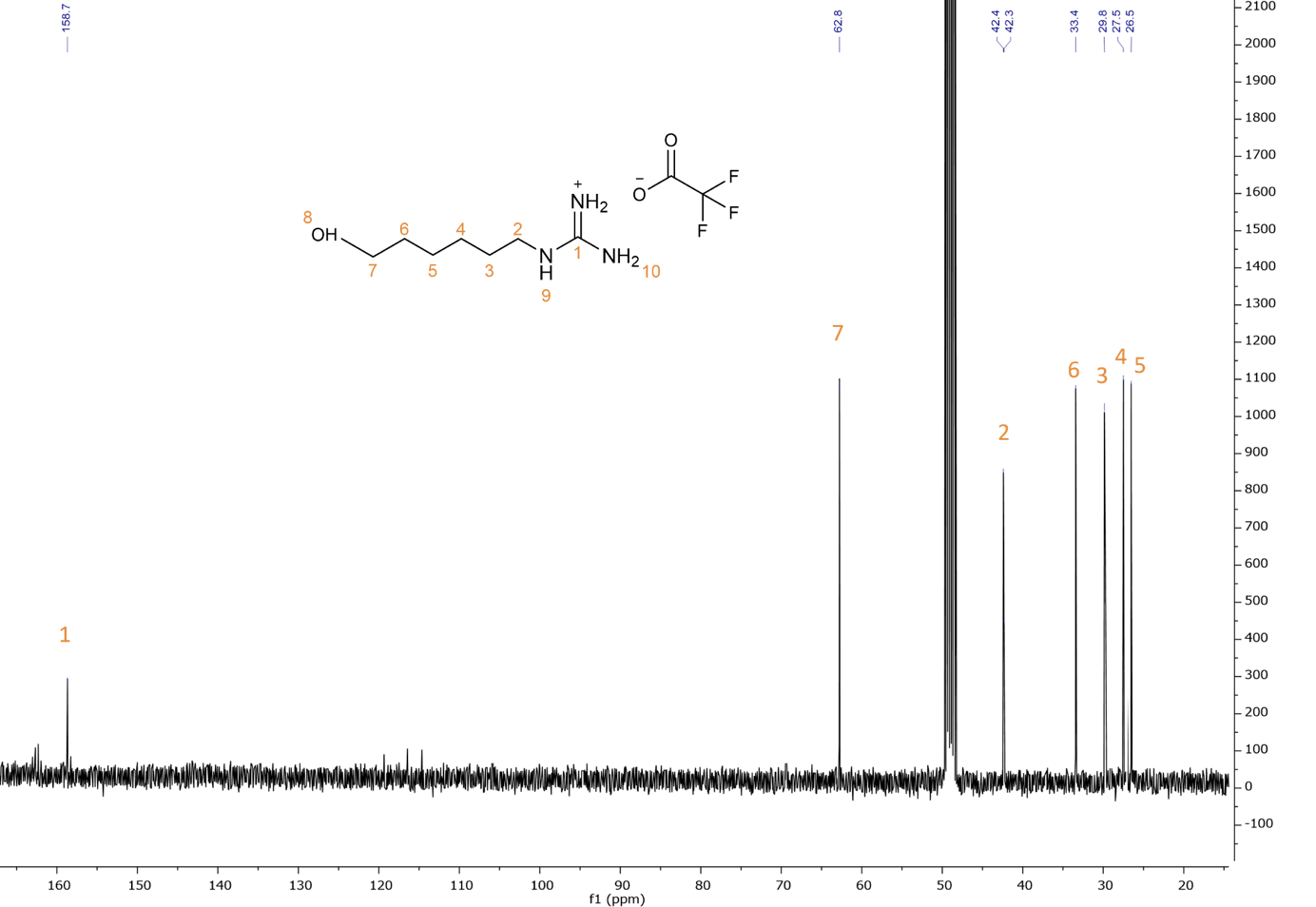

Supplement: S11 Fig — (DOCX) [file pone.0308049.s011.docx]

Figure S12. 1H NMR spectrum (400 MHz, methanol-*d4*) of *N*,*N*’-di-*tert*-butoxycarbonyl-7-hydroxyheptylguanidine (**7**).


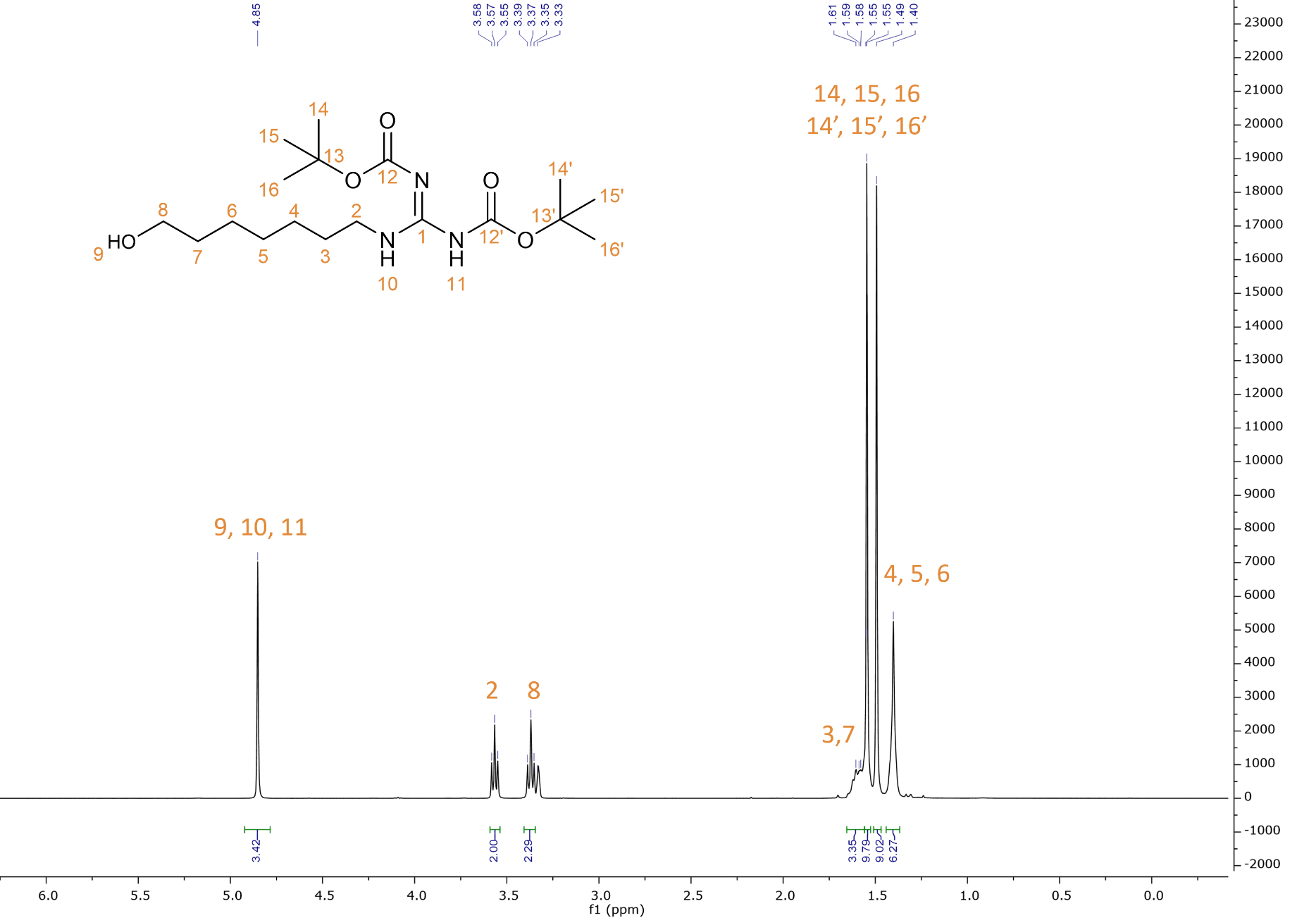

Supplement: S12 Fig — (DOCX) [file pone.0308049.s012.docx]

Figure S13. 13C NMR spectrum (101 MHz, methanol-*d4*) of *N*,*N*’-di-*tert*-butoxycarbonyl-7-hydroxyheptylguanidine (**7**).


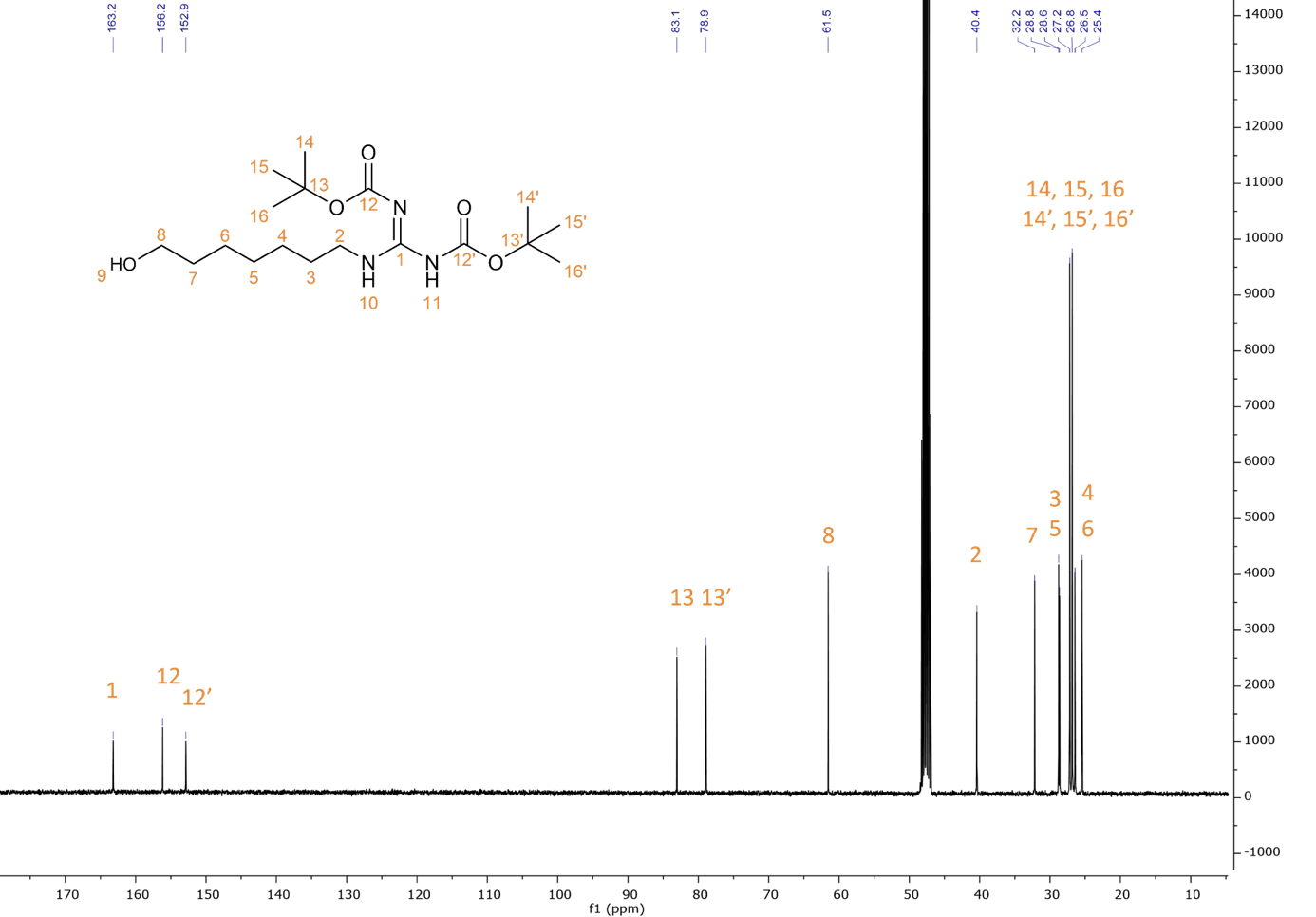

Supplement: S13 Fig — (DOCX) [file pone.0308049.s013.docx]

Figure S14. 1H NMR spectrum (400 MHz, methanol-*d4*) of 7-hydroxyheptylguanidine (**3**).


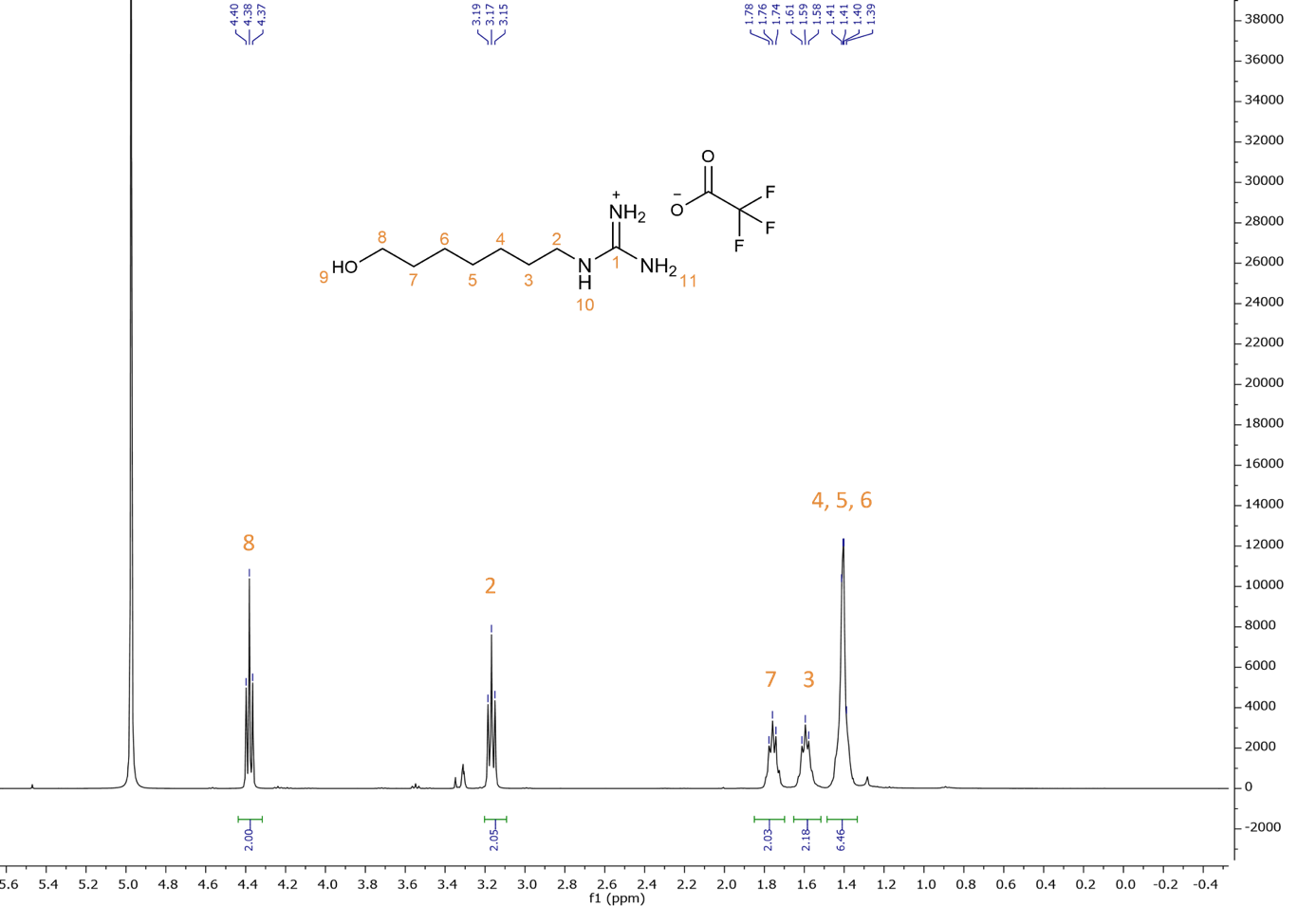

Supplement: S14 Fig — (DOCX) [file pone.0308049.s014.docx]

Figure S15. 13C NMR spectrum (101 MHz, methanol-*d4*) of 7-hydroxyheptylguanidine (**3**).


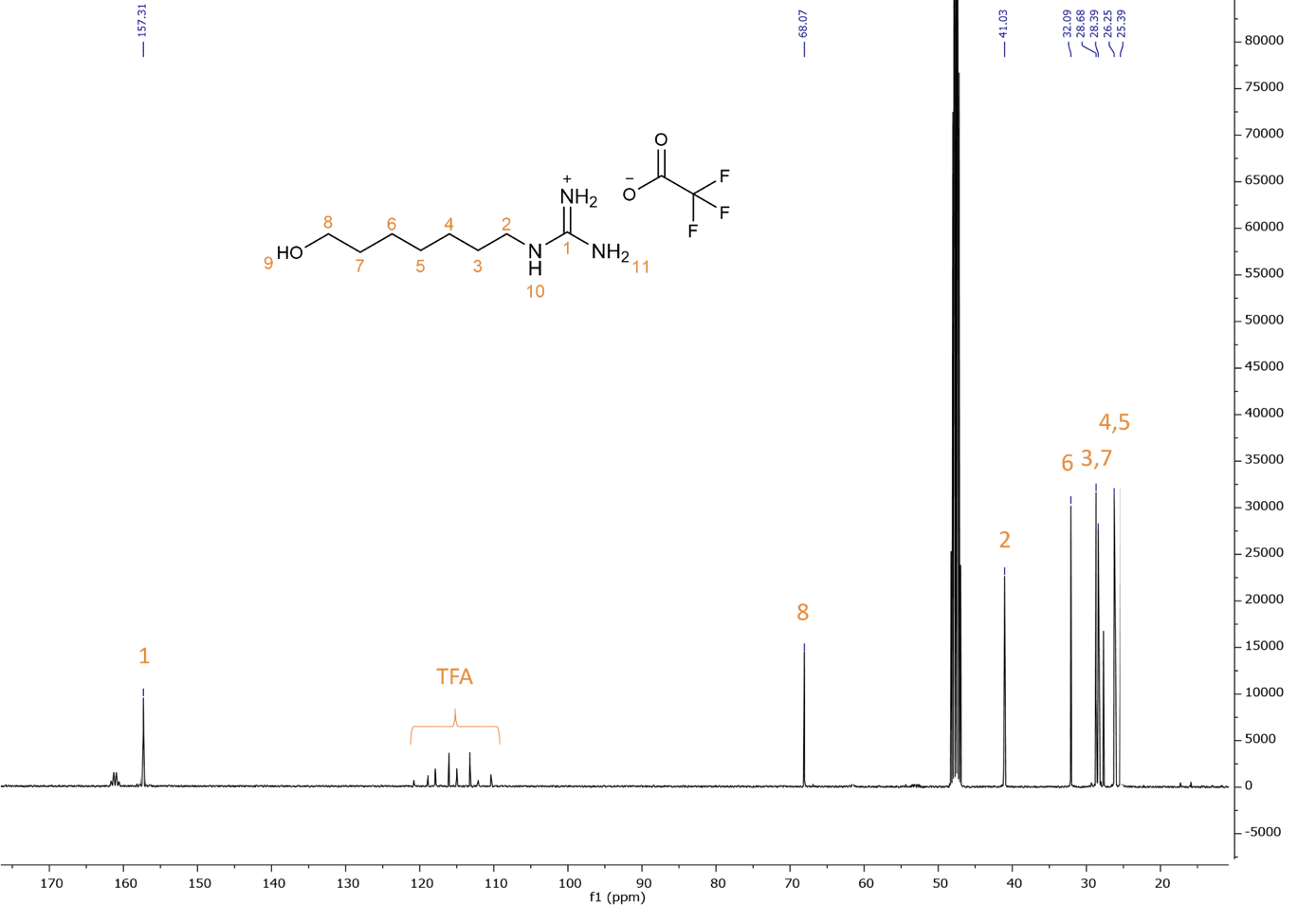

Supplement: S15 Fig — (DOCX) [file pone.0308049.s015.docx]

Figure S16. 1H NMR spectrum (400 MHz, methanol-*d4*) of *N*,*N*’-di-*tert*-butoxycarbonyl-8-hydroxyoctylguanidine (**8**).


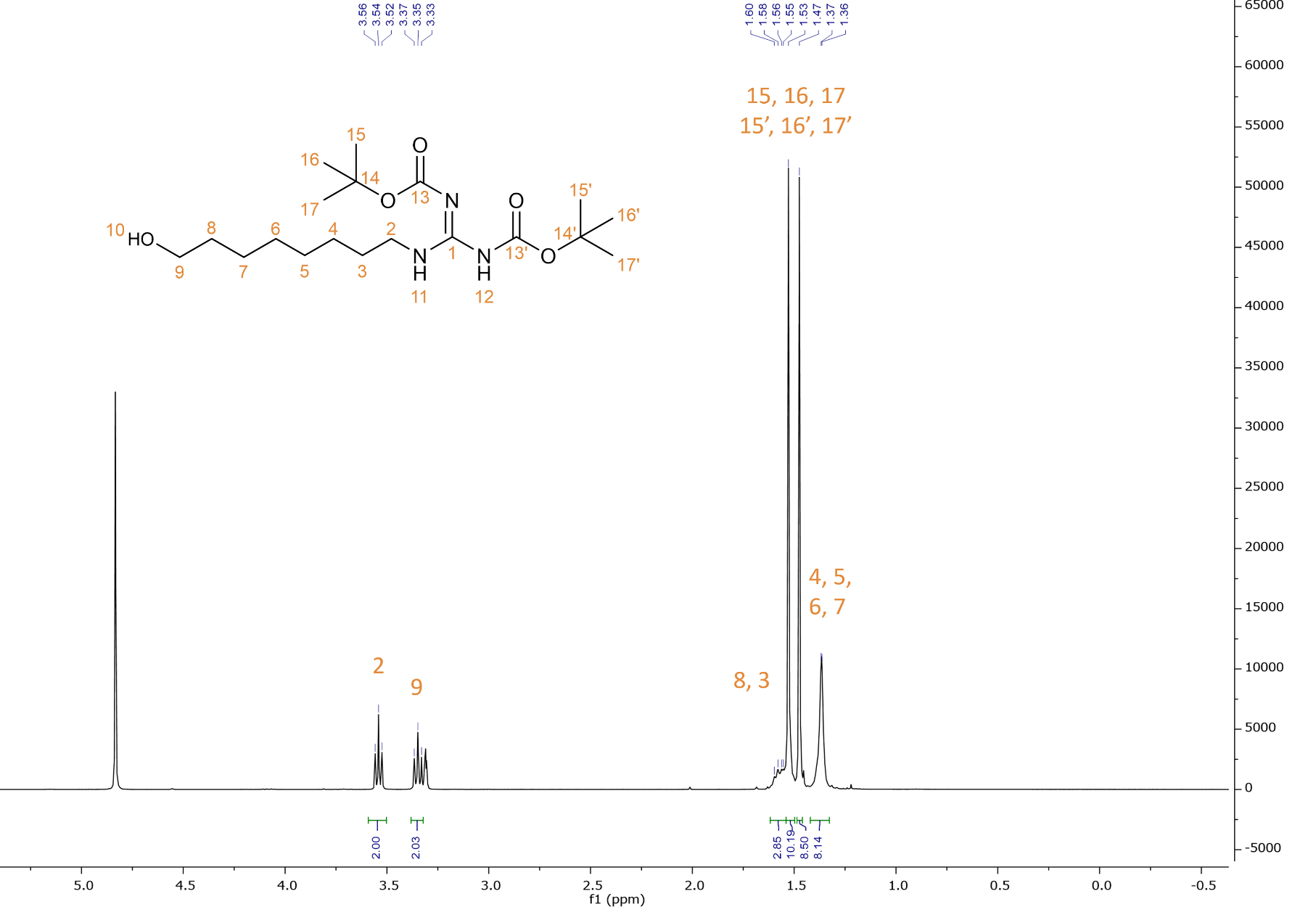

Supplement: S16 Fig — (DOCX) [file pone.0308049.s016.docx]

Figure S17. 13C NMR spectrum (101 MHz, methanol-*d4*) of *N*,*N*’-di-*tert*-butoxycarbonyl-8-hydroxyoctylguanidine (**8**).


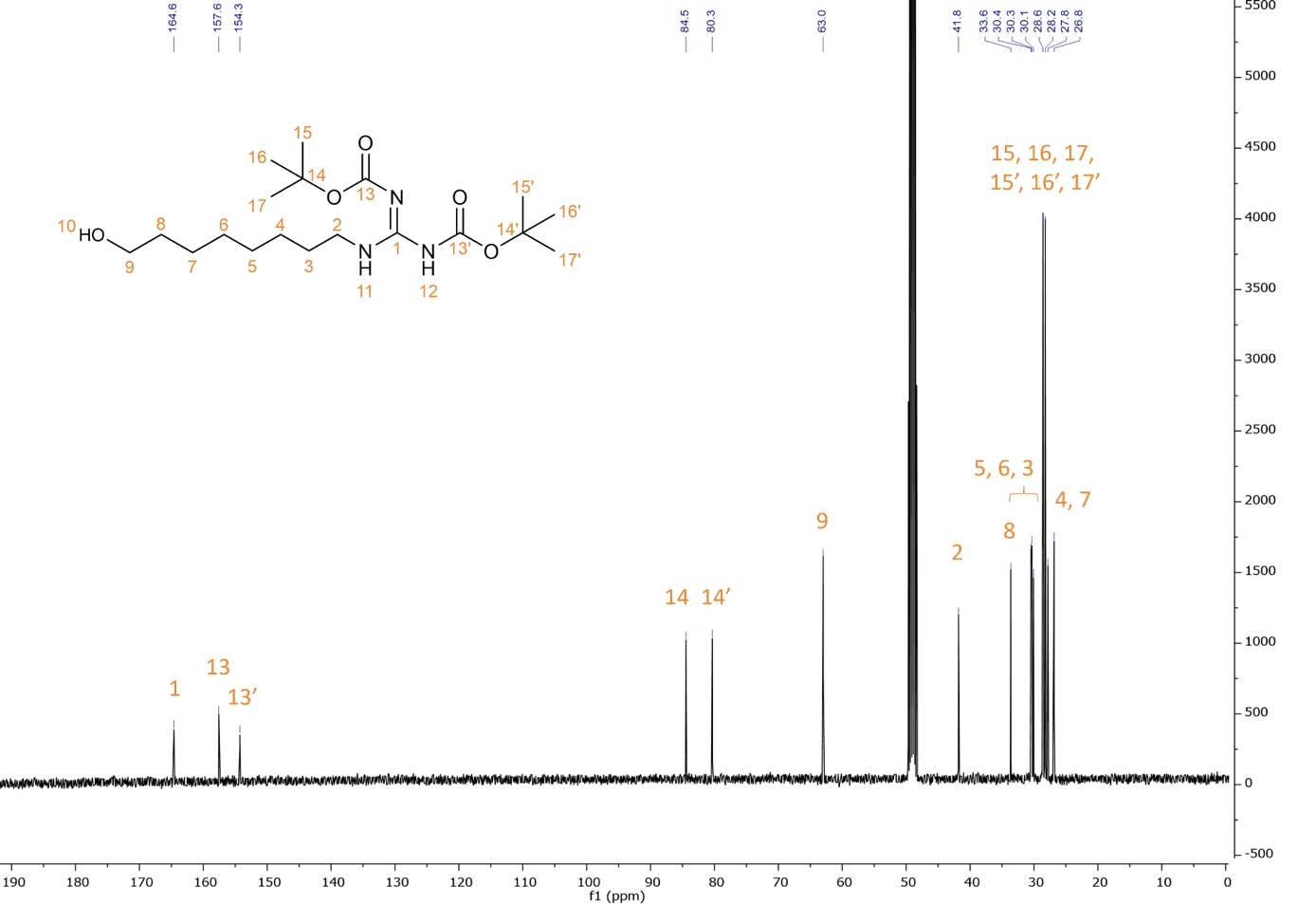

Supplement: S17 Fig — (DOCX) [file pone.0308049.s017.docx]

Figure S18. 1H NMR spectrum (400 MHz, methanol-*d4*) of 8-hydroxyoctylguanidine (**4**).


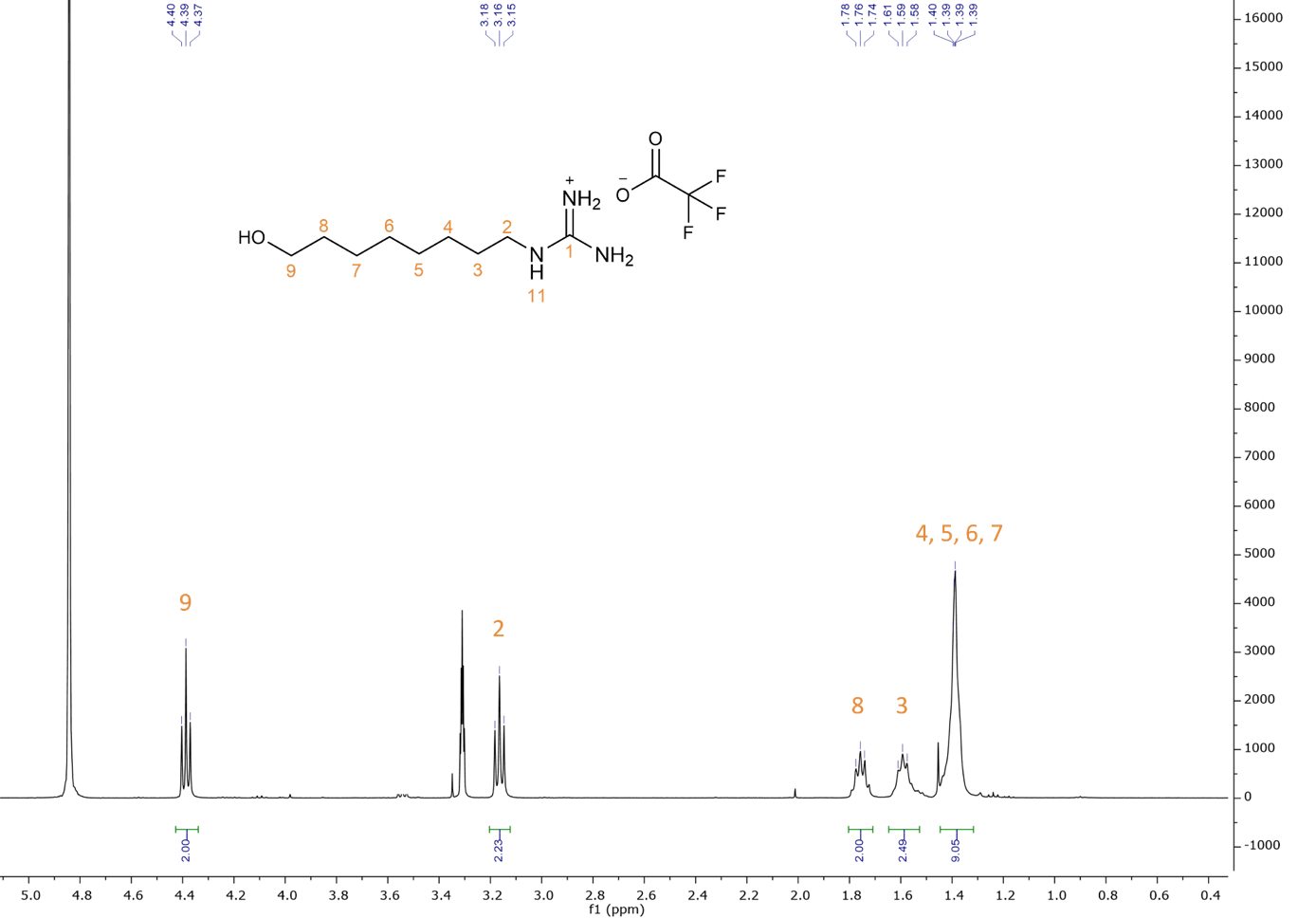

Supplement: S18 Fig — (DOCX) [file pone.0308049.s018.docx]

Figure S19. 13C NMR spectrum (101 MHz, methanol-d4) of 8-hydroxyoctylguanidine (**4**).


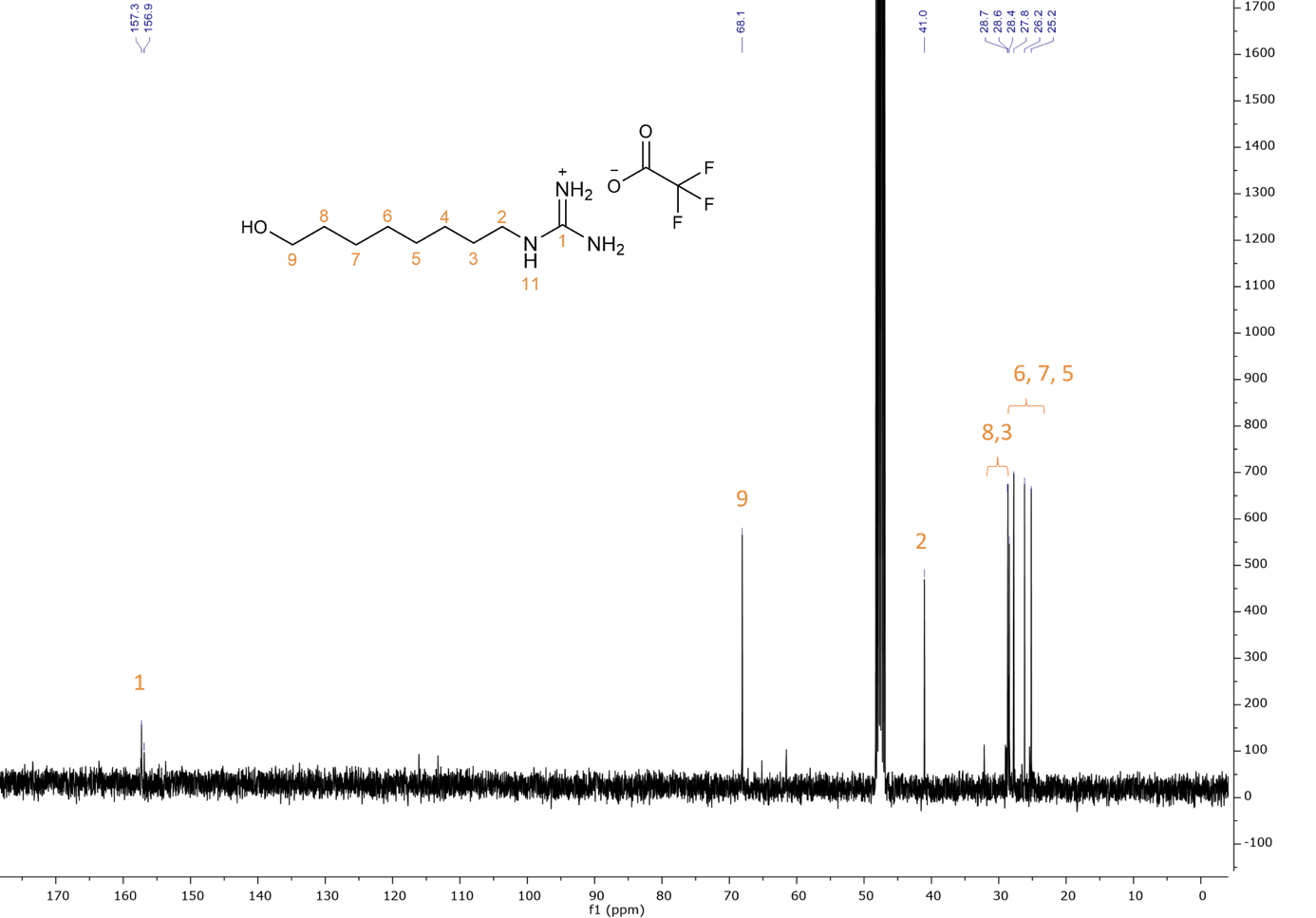

Supplement: S19 Fig — (DOCX) [file pone.0308049.s019.docx]
