# Supplementary material for: Development of a reliable, sensitive, and convenient assay for the discovery of new eIF5A hypusination inhibitors
Source: PLoS One. 2025 Feb 12;20(2):e0308049. doi: 10.1371/journal.pone.0308049 (PMC11819603; doi:10.1371/journal.pone.0308049)

Figure S3. Reaction of hypusination of eIF5A performed at various substrate concentration of spermidine or spermine.


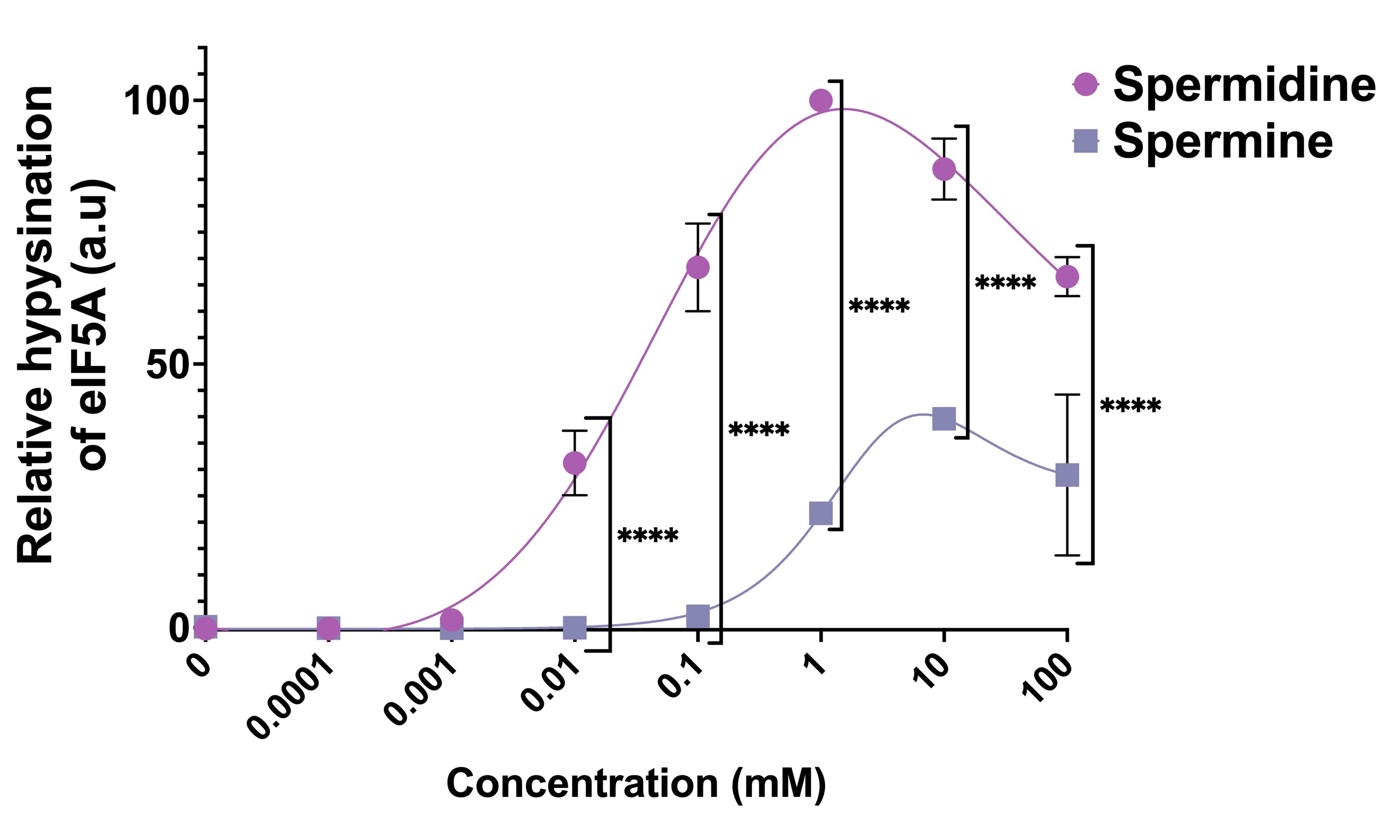

Supplement: S3 Fig — (DOCX) [file pone.0308049.s003.docx]
