# Supplementary material for: Development of a reliable, sensitive, and convenient assay for the discovery of new eIF5A hypusination inhibitors
Source: PLoS One. 2025 Feb 12;20(2):e0308049. doi: 10.1371/journal.pone.0308049 (PMC11819603; doi:10.1371/journal.pone.0308049)
Supplement: S20 Raw images — (PDF) [file pone.0308049.s020.pdf]

Original Western Blot from Figure 1B.

Image taken with Fuji LAS-4000 imager.

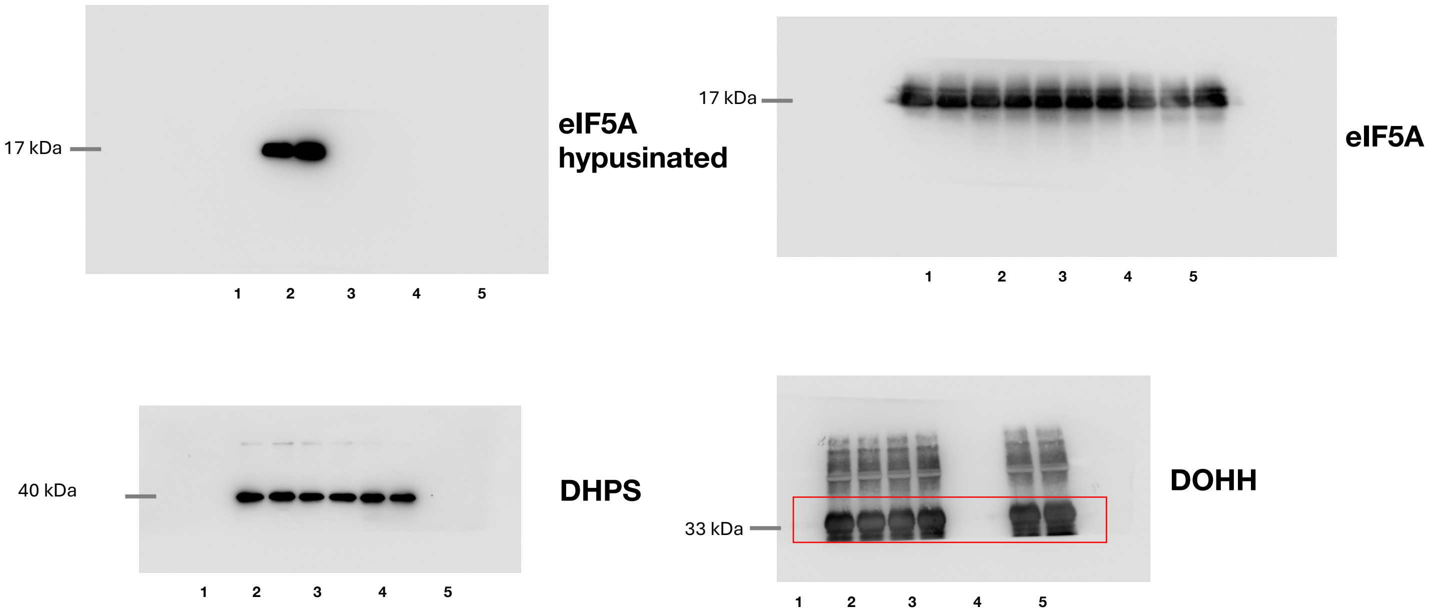

Quantification from Figure 2B

eIF5A (µg)	Relative hypusination of eIF5A (a.u)						Mean	Std. Deviation
0	-1,3217911	-1,0073562	0,29696629	1,07723064	0,6812756	0,27367481	0,07723	0,01476
1	9,21760059	10,7199006	8,27429592	5,46767338	8,57708507	8,1112556	8,395	1,719
2	45,5173619	35,4205081	38,8676462	35,3739252	25,0674482	31,6007065	35,31	6,863
4	80,4312804	73,6651074	76,9026222	89,6896411	94,0684381	87,1042876	83,64	7,906
6	101,05588	103,198696	91,5413133	106,401273	102,278682	95,5241552	100,0	5,461

Quantification from Figure 2C

Quantity of proteins (µg)	DHPS			Mean	Std. Deviation	DOHH			Mean	Std. Deviation
0	-0,0391546	-0,0414281	-0,0351128	-0,03857	0,003199	-0,0391546	-0,0414281	-0,0351128	-0,03857	0,003199
0,25	38,6746379	37,7652408	34,8097002	37,08	2,021	94,1731223	105,970024	99,8568542	100,0	5,900
0,5	80,759515	70,6045807	64,7187605	72,03	8,115	127,416639	125,042102	122,819131	125,1	2,299
1	82,5277871	123,223308	91,3691479	99,04	21,40	114,71034	122,490738	126,608286	121,3	6,042
2	99,9578983	93,8952509	109,910744	101,3	8,086	99,9578983	93,8952509	109,910744	101,3	8,086

Quantification from Figure 2D

Spermidine (µM)	pH: 8.0			Mean	Std. Deviation	pH: 9.0			Mean	Std. Deviation
0,01	0,59862668	-1,10922	0,51059334	6,66666662615019e-009	0,961620629266078	-0,5282	0,66905335	-0,1408533	1,66666666763978e-008	0,610928505337209
0,1	0,12324667	1,90152004	1,63742004	1,22072891666667	0,959576814961359	-3,8030401	1,25007336	3,08116674	0,176066666666667	3,56555658165304
1	10,1942602	17,7651271	22,3428605	16,7674159333333	6,13544562288336	9,38435354	15,1417337	17,8883737	14,1381536466667	4,33992735112098
10	67,5039615	74,9691883	65,3207348	69,2646282	5,05945887983905	41,2348142	49,6684078	39,2452609	43,3828276333333	5,53362169894909
100	112,330536	92,3821821	96,9070955	100,539937866667	10,4586023720045	44,9674277	46,6048477	50,9360878	47,5027877333333	3,08398250237092
1000	94,9175421	105,481542	99,6009155	99,9999998666667	5,29329526104376	41,2700276	50,5311345	45,1963143	45,6658254666667	4,64837130670791

Quantification from Figure 2E

DMSO (%)	0	0.01	0.1	1	10
Relative hypusination of eIF5A (a.u)	101,688625	85,3219521	91,0001856	84,9322694	59,4358879
	106,865838	103,470032	89,5527927	95,5650399	70,1243273
	91,4455372	102,412321	86,1013175	89,3301169	72,0170718
Mean	100,0	97,07	88,88	89,94	67,19
Std. Deviation	7,848	10,19	2,517	5,343	6,784

Quantification from Figure 3A

GC7 (µM)	Spermidine 100 µM			Mean	Std. Deviation	Spermidine 5 µM			Mean	Std. Deviation
0						47.6267028	55.2669431	55.8507488	52.9147982333333	4.58891841917774
1	95.4141636	98.3585752	106.227261	99.9999999333333	5.59030188915326	51.7894915	55.7999831	54.4800745	54.0231830333333	2.04391122058965
10	91.1752263	87.2662662	107.01413	95.1518741666667	10.457285850422	21.4823589	20.7208732	16.5073187	19.5701836	2.67970542496095
100	85.159489	87.3170319	102.92749	91.8013369666667	9.69573147483982	16.8372959	17.4718673	16.0758101	16.7949911	0.69898941229123
1000	60.0812251	49.1665962	46.4506303	51.8994838666667	7.21455368677861	-1.3368305	2.80057534	0.05922667	0.50765717	2.10483947278984
10000	-3.9851087	3.40130299	0.81225146	0.0761485833333334	3.74782003267498	-1.8698705	-2.0221677	-2.2252306	0	0

Quantification from Figure 3B

	Spermine												Mean	SD
0	95,3	98,0	104,7	88,5	100,2	92,1	106,7	106,8	105,9	92,1	103,6	106,0	100,0	6,6
1	99,2	116,9	102,2										106,1	9,5
10	115,6	117,6	109,0										114,0	4,5
100	113,3	99,4	100,9										104,5	7,6
1000	120,5	106,1	107,6										111,4	7,9

	Putrescine												Mean	SD
0	95,3	98,0	104,7	88,5	100,2	92,1	106,7	106,8	105,9	92,1	103,6	106,0	100,0	6,6
1	117,0	110,3	106,7										111,3	5,2
10	107,4	91,1	85,3										94,6	11,5
100	99,3	101,3	85,4										95,3	8,7
1000	29,4	28,9	33,2										30,5	2,4

	BENSPM												Mean	SD
0	95,3	98,0	104,7	88,5	100,2	92,1	106,7	106,8	105,9	92,1	103,6	106,0	100,0	6,6
1	93,1	102,9	95,9										97,3	5,0
10	96,4	108,4	100,8										101,9	6,1
100	80,9	94,3	90,2										88,5	6,9
1000	37,6	43,2	48,0										42,9	5,2

Quantification from Figure 3C

	CPX						Mean	SD	DFX						Mean	SD
0	106,47	106,08	107,02	90,17	93,76	96,53	100,01	7,43	106,47	106,08	107,02	90,17	93,76	96,53	100,01	7,43
1	100,95	94,61					97,78	4,48	32,62	24,92	27,33				28,29	3,94
10	86,73	87,91	80,89				85,18	3,76	55,19	64,72	55,35				58,42	5,46
100	53,86	55,41	55,79				55,02	1,02	33,78	35,48	33,38				34,21	1,11

	EDTA						Mean	SD	Folate						Mean	SD
0	106,47	106,08	107,02	90,17	93,76	96,53	100,005	7,425846	106,47	106,08	107,02	90,17	93,76	96,53	100,01	7,43
1	50,50	62,61	56,30				56,47243	6,055153	51,66	59,55	57,93				56,38	4,17
10	36,35	36,12	28,22				33,5642	4,630802	61,33	52,22	58,41				57,32	4,65
100	13,28	12,17	14,12				13,19077	0,981921	73,36	59,37	63,24				65,33	7,22

	Spermidine						Mean	SD	Spermine						Mean	SD
0	87,30	90,49	92,49	111,14	118,43	100,15	100,00	12,43	87,30	90,49	92,49	111,14	118,43	100,15	100,00	12,43
1	78,70	85,14	90,64				84,83	5,98	68,73	67,11	72,30				69,38	2,66
10	83,50	80,95	83,31				82,59	1,42	58,61	57,72	61,03				59,12	1,71
100	54,37	76,21	71,30				67,29	11,46	47,30	55,06	60,60				54,32	6,68

Quantification from Figure 3D

	CTRL	GC7- DHPS	GC7- DOHH
Relative hypusination of eIF5A (a.u)	119,345019	7,89335573	103,327385
	118,484308	3,04398027	99,8215598
	107,714916	1,74241629	95,5809804
	95,6859452		
	80,4870368		
	78,2827753		
Mean	100,0	4,227	99,58
SD	18,15	3,242	3,879

Quantification from Figure 3E

Speermidine (µM)	pH: 8.0			Mean	SD	pH: 9.0			Mean	SD
1	2,57893468	1,30370955	-3,8826442	0,00	3,42	2,25684174	-1,9829532	-0,2738886	0,00	2,13
10	41,0460352	51,182103	59,1818401	50,47	9,09	41,1906483	54,9683385	57,2492824	51,14	8,69
100	82,4974254	92,9555862	86,4282741	87,29	5,28	86,730647	87,2236464	83,720064	85,89	1,90
1000	97,25454107	98,7664059	98,14194	98,05	0,76	80,08501501	102,177962	113,096255	98,45	16,82
10000	106,411183	95,2102369	98,3785797	100,00	5,77	102,920748	112,701856	105,195118	106,94	5,12

Quantification from Figure 3F

	CTRL	GC7
Relative deoxyhypusination of eIF5A (a.u)	106,411183	8,66300326
	95,2102369	0,05911071
	98,3785797	13,8713138
Mean	100,0	7,531
SD	5,774	6,975

Quantification from Figure 3G

	CTRL	CPX	EDTA
Relative deoxyhypusination of eIF5A (a.u)	94,8746166	104,153691	96,237327
	106,050247	101,72329	92,4161184
	99,0751364	97,7545716	104,223934
Mean	100,0	101,2	97,63
SD	5,645	3,230	6,025

Quantification from Figure 5A

	1										Mean	SD
0	76,90	83,55	80,18	88,29	77,19	121,16	116,62	109,38	123,71	108,02	98,5	19,1
1	64,13	73,17	74,83								70,7	5,8
10	58,75	64,57	64,08								62,5	3,2
100	65,88	58,80	59,78								61,5	3,8
1000	57,04	54,69	53,76								55,2	1,7

	2										Mean	SD
0	76,90	83,55	80,18	88,29	77,19	121,16	116,62	109,38	123,71	108,02	98,5	19,1
1	82,75	74,49	86,56								81,3	6,2
10	59,19	72,14	61,73								64,4	6,9
100	56,21	59,04	62,17								59,1	3,0
1000	48,73	44,87	40,13								44,6	4,3

	3										Mean	SD
0	76,90	83,55	80,18	88,29	77,19	121,16	116,62	109,38	123,71	108,02	98,5	19,1
1	70,28	76,78	80,01								75,7	5,0
10	66,23	69,11	61,58								65,6	3,8
100	63,39	63,05	64,22								63,6	0,6
1000	65,00	52,59	57,09								58,2	6,3

	4										Mean	SD
0	76,90	83,55	80,18	88,29	77,19	121,16	116,62	109,38	123,71	108,02	98,5	19,1
1	113,59	112,32	102,25								109,4	6,2
10	72,73	71,95	116,08								86,9	25,3
100	64,32	69,55	67,69								67,2	2,7
1000	86,31	52,49	53,13								64,0	19,3

Original Western Blot from Figure 5B.

Image taken with Fuji LAS-4000 imager.

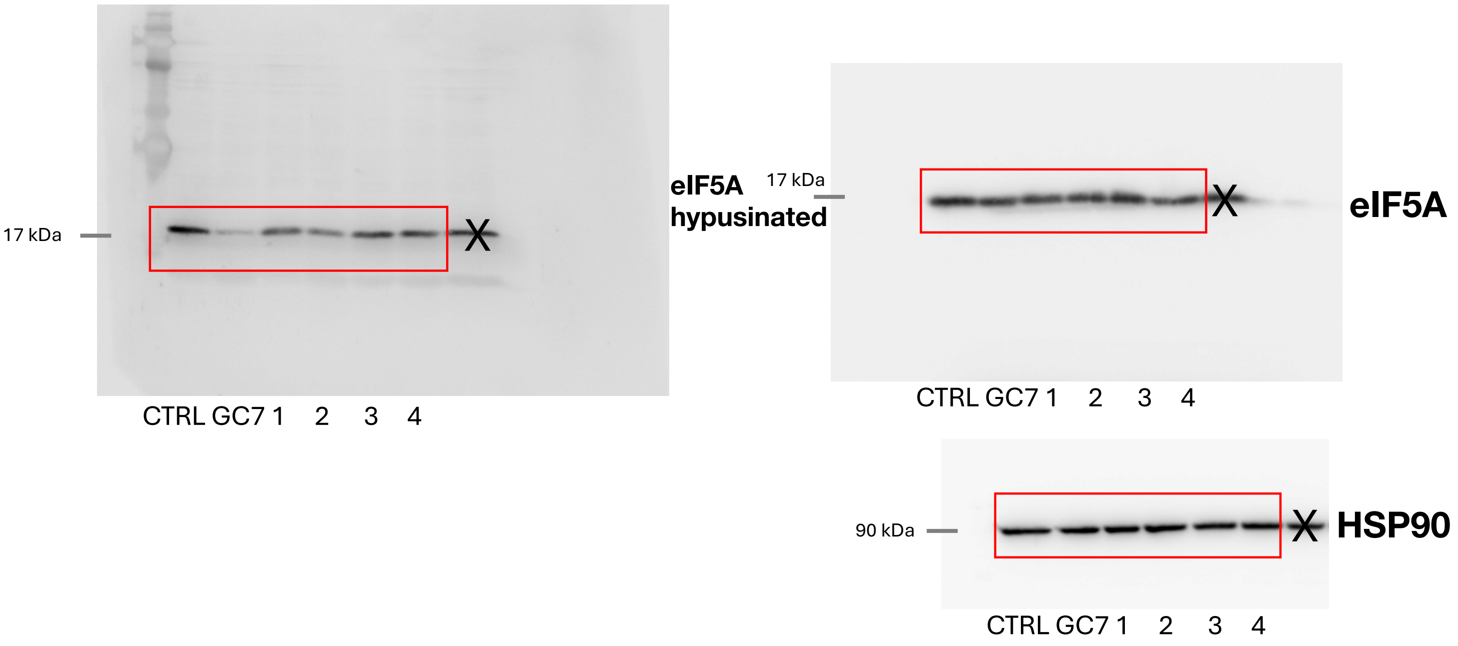

Original Western blots and quantifications from Figure 5B

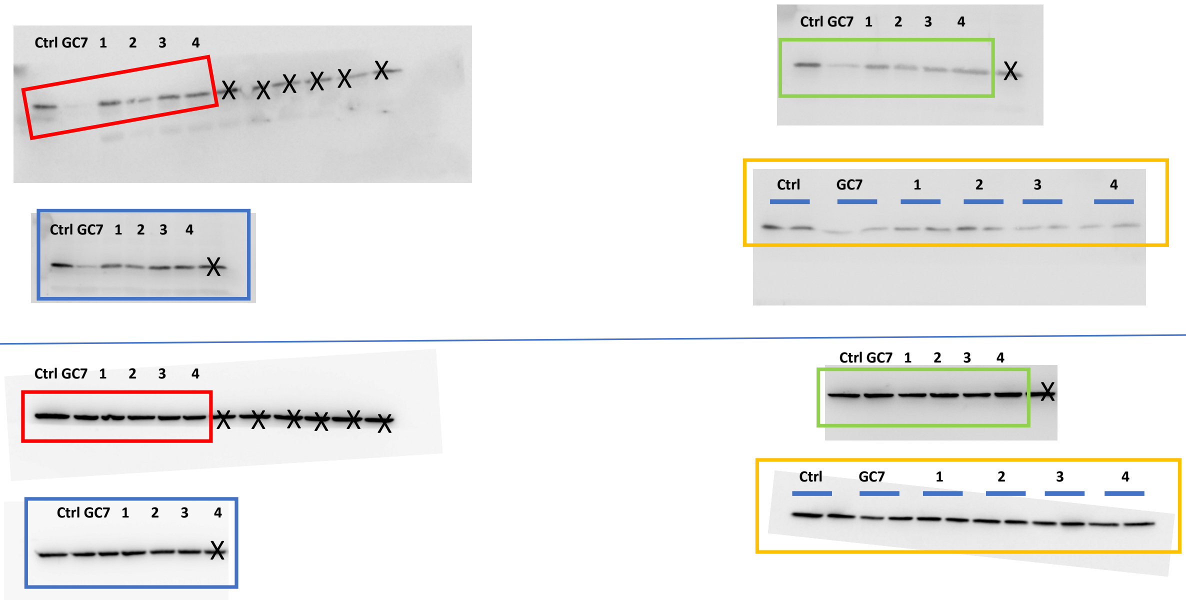

eIF5A hypusinated

HSP90

	Ctrl	GC7	1	2	3	4
	100	23	69	43	72	66
	100	20.8	53	35	39	74
	100	8	70	34	62	81
	100	30	68	66	29	38
Mean	100	20.45	65	44.5	50.5	64.75
Std. Deviation	0	9.18	8.042	14.89	19.91	18.86

Figure S1. Original Western Blot from Figure 5C

Image taken with Fuji LAS-4000 imager.

.

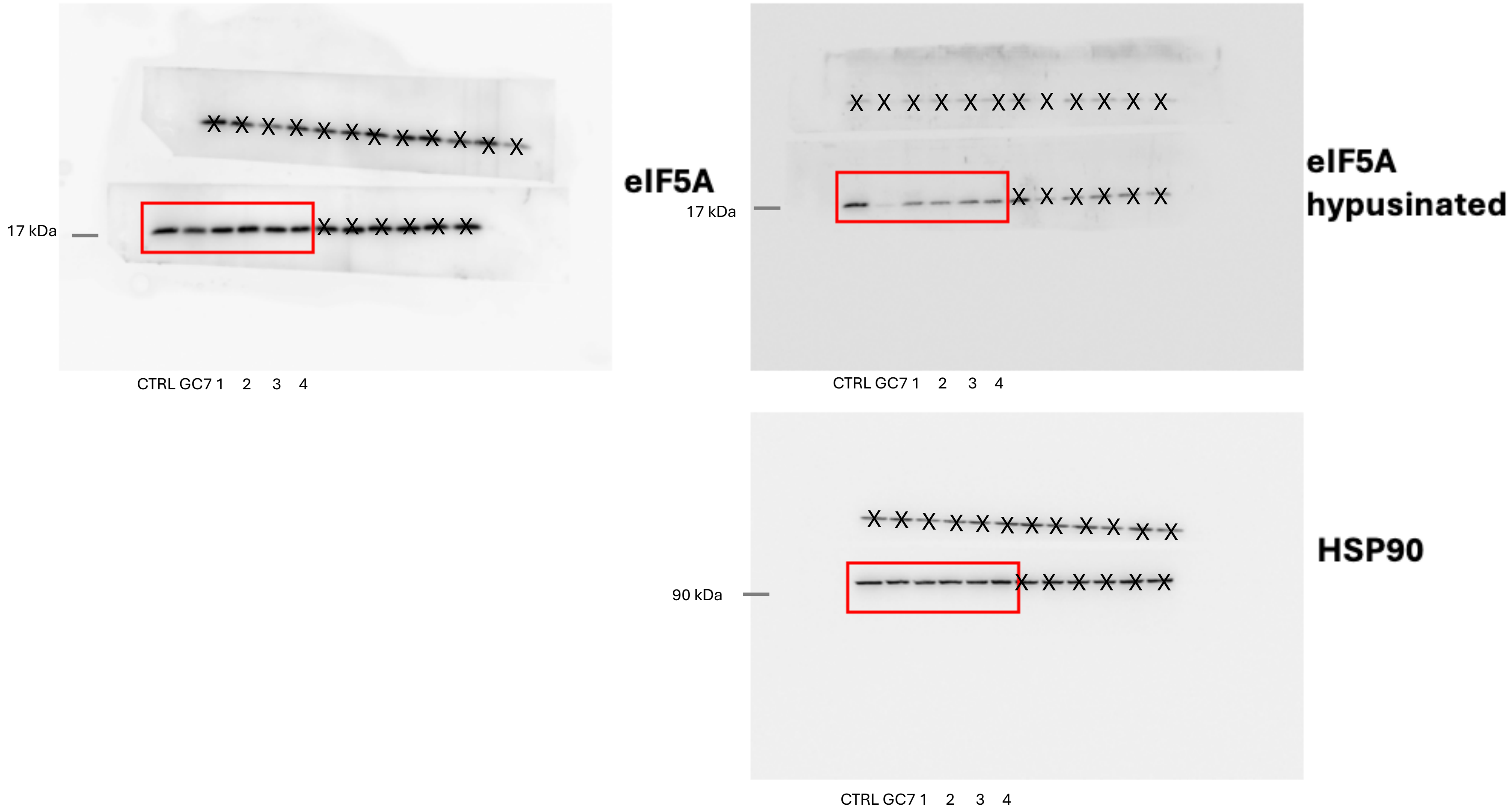

Original Western blots and quantifications from Figure 5C

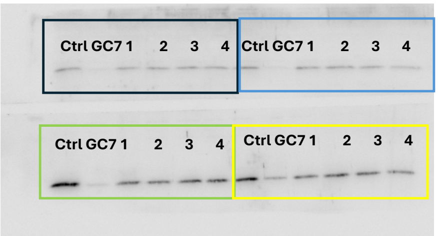

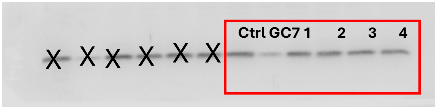

eIF5A hypusinated

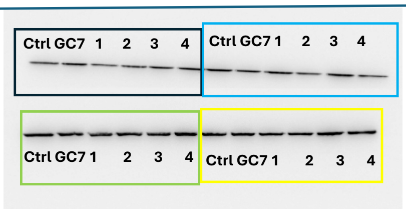

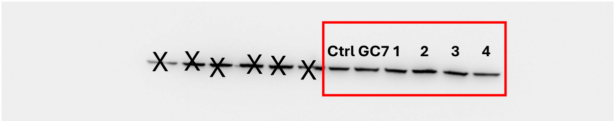

HSP90

	Ctrl	GC7	1	2	3	4
	100,003675	24,8147235	70,5214393	80,4130928	80,4475855	84,2801048
	100,000815	27,3465284	72,4569867	80,8357008	55,3669599	44,743274
	100,001024	7,5883067	41,6787127	36,5704607	43,816677	44,5821989
	100,003233	31,355503	51,2725671	68,1145575	53,5399569	43,6910603
	100,000403	21,5342164	68,0394437	71,3884128	73,387264	75,7501855
Mean	100	22.53	60.79	67.46	61.31	58.61
Std. Deviation	0.001507	9.091	13.59	18.14	15.11	19.78

Quantification from Figure S2

pH	7.5	8.0	9.0
Relative hypusination of eIF5A (a.u)	41,24916	106,9305	38,54375
	43,00267	97,56179	30,97862
	58,88444	95,50768	43,95458
Mean	47,71	100,0	37,83
SD	9,715	6,089	6,518

Quantification from Figure S3

Concentration (mM)	Spermidine			Mean	SD	Spermine			Mean	SD
0,0001	-0,281854	-0,1342162	-0,1476378	-0,19	0,08	0,81200787	-0,1610594	-0,0805297	0,19	0,54
0,001	-0,1610594	-0,2617215	-0,2013243	-0,21	0,05	-0,1811918	-0,1140838	0,06039728	-0,08	0,12
0,01	1,41598067	1,23478883	1,72467788	1,46	0,25	-0,0671081	-0,3355404	0,18790265	-0,07	0,26
0,1	24,366947	33,0306013	36,2585004	31,22	6,15	-0,140927	-0,0067108	0,16777022	0,01	0,15
1	76,82534	67,9335183	60,2026664	68,32	8,32	3,22118826	1,77165354	1,50322119	2,17	0,92
10	99,4273443	100,165533	100,407122	100,00	0,51	20,9444345	20,4545455	23,7159986	21,70	1,76
100	90,7502684	89,8375984	80,301539	86,96	5,79	39,8420723	39,1038833	39,9695777	39,64	0,47
1000	65,9269864	70,5909986	63,2493737	66,59	3,72	31,0643343	12,7706693	43,083393	28,97	15,26
